# Supplementary material for: A vision transformer for decoding surgeon activity from surgical videos
Source: Nat Biomed Eng. Author manuscript; Available in PMC 2023 Jul 10. (PMC10307635; doi:10.1038/s41551-023-01010-8)
Supplement: supp [file NIHMS1899164-supplement-supp.pdf]

nature biomedical engineering

Article https://doi.org/10.1038/s41551-023-01010-8

A vision transformer for decoding surgeon activity from surgical videos

In the format provided by the authors and unedited

nature biomedical engineering

Article https://doi.org/10.1038/s41551-023-01010-8

A vision transformer for decoding surgeon activity from surgical videos

In the format provided by the authors and unedited

nature biomedical engineering

Article https://doi.org/10.1038/s41551-023-01010-8

A vision transformer for decoding surgeon activity from surgical videos

In the format provided by the authors and unedited

Supplementary Note 1 - Dataset splits for training and evaluation

Sub-phase recognition

We use 10-fold Monte Carlo cross-validation in order to evaluate the performance of our deep learning framework. As such, in this section, we outline the training, validation, and test splits for each of those folds across the machine learning tasks described in the main manuscript (surgical sub-phase recognition, gesture classification, and skills assessment).

Fold	Training			Validation			Testing		
	n	v	s	n	v	s	n	v	s
0	3805	63	17	425	7	6	544	8	4
1	3853	63	18	379	7	5	542	8	6
2	3845	63	16	427	7	6	502	8	6
3	3771	63	18	455	7	6	548	8	6
4	3855	63	17	396	7	5	523	8	7
5	3854	63	16	455	7	6	465	8	6
6	3842	63	16	438	7	6	494	8	8
7	3827	63	16	449	7	5	498	8	6
8	3859	63	19	428	7	6	487	8	5
9	3818	63	17	488	7	4	468	8	6

Supplementary Table 1. Total number of video samples (n), videos (n), and surgeons (s), in the training, validation, and test sets of each fold for sub-phase recognition. These data splits are used for the 10-fold Monte Carlo cross-validation.

Gesture classification

Fold	Training			Validation			Testing		
	n	v	s	n	v	s	n	v	s
0	1161	66	10	36	11	5	44	12	5
1	1208	65	9	36	10	6	36	10	7
2	1206	65	10	44	11	7	32	12	7
3	1183	67	10	36	11	6	36	11	6
4	1173	65	10	40	11	7	36	10	6
5	1192	64	10	44	11	6	36	11	5
6	1187	66	10	40	10	5	28	11	7
7	1204	64	10	36	11	6	32	10	4
8	1211	66	10	44	11	7	52	12	6
9	1207	66	10	36	10	6	40	11	4

Fold	Training			Validation			Testing		
	n	v	s	n	v	s	n	v	s
0	1236	85	15	120	16	10	186	20	9
1	1308	82	15	90	18	10	114	20	10
2	1272	84	15	96	18	10	150	20	11
3	1224	85	15	78	16	12	198	20	9
4	1302	82	15	120	17	12	120	20	11
5	1176	84	15	132	18	7	222	19	9
6	1302	84	15	234	16	8	120	21	8
7	1326	86	15	126	19	11	96	20	8
8	1302	83	15	198	18	8	120	17	8
9	1176	85	15	102	18	10	216	21	10

Supplementary Table 2. Total number of videos samples (n), videos (v), and surgeons (s) in the training, validation, and test sets of each fold for gesture classification. These data splits are used for the 10-fold Monte Carlo cross-validation. Data are used for **(left)** suturing gesture classification and **(right)** dissection gesture classification.

Fold	Training			Testing		
	n	v	s	n	v	s
0	1364	33	7	14	5	3
1	1386	33	7	14	6	4
2	1390	33	7	14	6	2
3	1358	33	7	21	4	2
4	1318	34	6	14	5	3
5	1375	33	6	14	5	3
6	1367	32	7	14	6	3
7	1310	33	7	14	5	3
8	1385	33	7	14	4	2
9	1411	34	7	14	5	3

Fold	Training			Testing		
	n	v	s	n	v	s
0	691	34	7	102	5	1
1	697	34	7	96	5	1
2	679	34	7	114	5	1
3	701	34	7	92	5	1
4	703	34	7	90	5	1
5	677	34	7	116	5	1
6	707	35	7	86	4	1
7	696	34	7	97	5	1

Supplementary Table 3. Total number of videos samples (n), videos (v), and surgeons (s) in the training and test sets of each fold for gesture classification in external datasets. (left) DVC UCL dataset and **(right)** JIGSAWS dataset. These data splits are used for 10-fold cross-validation.

Skill assessment

Fold	Training			Validation			Testing		
	n	v	s	n	v	s	n	v	s
0	748	63	17	82	7	6	82	8	4
1	752	63	18	82	7	5	78	8	6
2	778	63	16	44	7	6	90	8	6
3	730	63	18	102	7	6	80	8	6
4	728	63	17	60	7	5	124	8	7
5	774	63	16	46	7	6	92	8	6
6	724	63	16	102	7	6	86	8	8
7	752	63	16	102	7	5	58	8	6
8	754	63	19	86	7	6	72	8	5
9	756	63	17	90	7	4	66	8	6

Fold	Training			Validation			Testing		
	n	v	s	n	v	s	n	v	s
0	442	63	17	42	7	6	46	8	4
1	438	63	18	42	7	5	50	8	6
2	432	63	16	44	7	6	54	8	6
3	452	63	18	42	7	6	36	8	6
4	438	62	17	38	7	5	54	8	7
5	448	63	16	30	7	6	52	8	6
6	400	63	16	62	7	6	68	8	8
7	450	63	16	54	7	5	26	8	6
8	408	63	19	48	7	6	74	8	5
9	412	63	17	58	7	4	60	8	6

Supplementary Table 4. Total number of videos samples (n), videos (v), and surgeons (s) in the training, validation, and test sets of each fold for skill assessment. These data splits are used for the 10-fold Monte Carlo cross-validation. Data are used for **(left)** needle handling skill assessment and **(right)** needle driving skill assessment.

Supplementary Note 2 - Duration of video samples

In this section, we present the distribution of the duration of video samples used for training and evaluating SAIS' ability to decode surgical sub-phases and the skill-level of surgeons (Fig. 1). These are shown for the three suturing sub-phases of needle handling, needle driving, and needle withdrawal (columns) for the different hospitals: USC, SAH, and HMH (rows). As we can see, the video samples can span $5-100$ seconds.

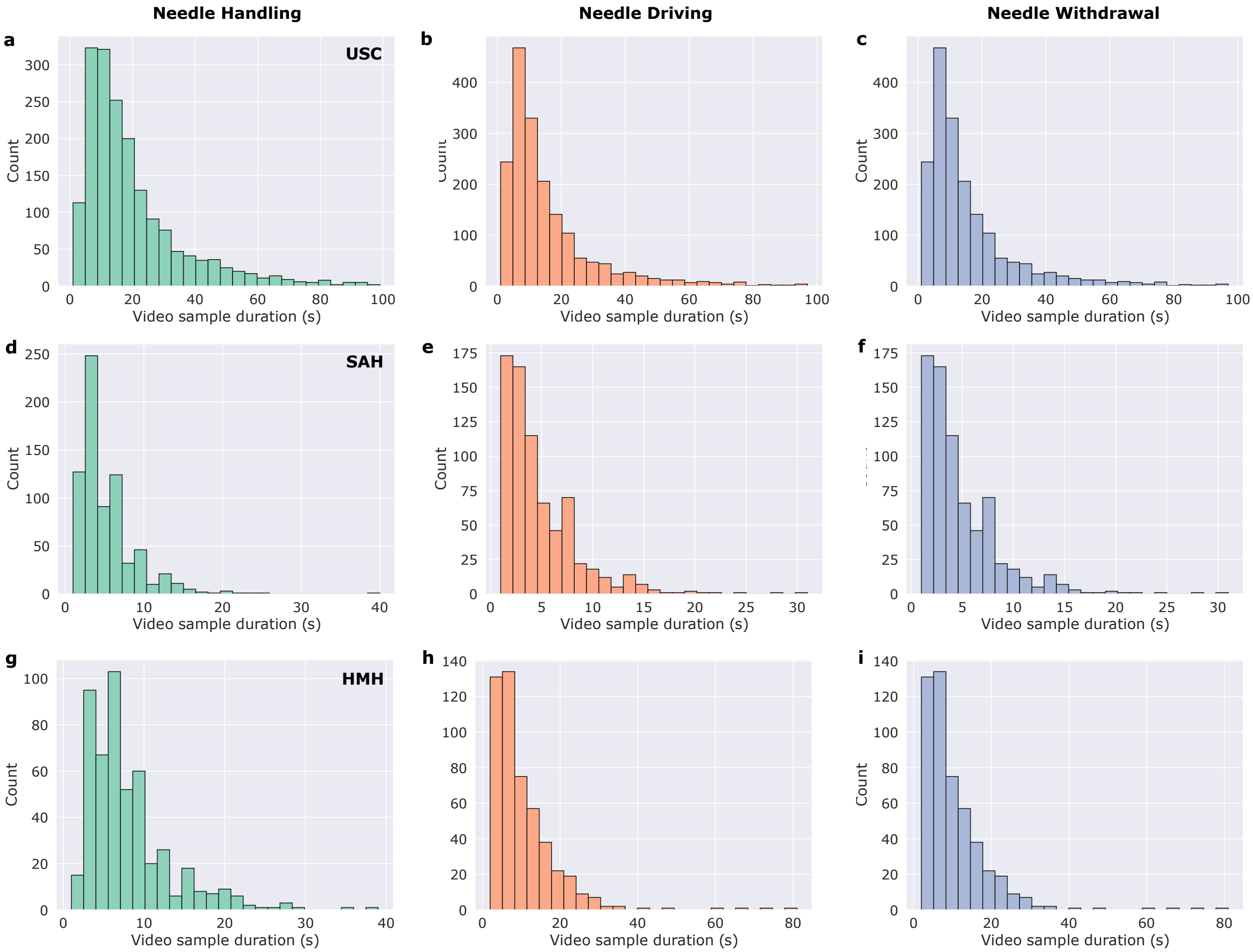

Supplementary Figure 1. Distribution of the duration of video samples for the three sub-phases and across hospitals. Each row reflects a different hospital. Each column reflects a different suturing sub-phase: needle handling, needle driving, and needle withdrawal. We see that video samples can span $5-100$ seconds.

Supplementary Note 3 - Distinguishing between tissue dissection and suturing

In this main manuscript, we claimed that SAIS can reliably distinguish between the surgical activities of tissue dissection and tissue suturing. Here, we provide evidence in support of that claim and details of the experiments conducted.

Throughout the manuscript, we trained and evaluated SAIS using 10-fold Monte Carlo cross-validation. We adopt the same strategy to distinguish between video samples of the nerve-sparing (NS) dissection step and the vesico-urethral anastomosis (VUA) suturing step (Table 5). We balance the number of samples from each category (NS and VUA) in each data split (training, validation test), such that 50% of the samples are from each category. We also follow the same implementation details outlined in the Methods section as it pertains to the video samples used, the frames selected in each video sample, and so forth. Video samples of the VUA step consisted of a subset of those used for the sub-phase recognition task (Table 1). Video samples of the NS step consisted of a subset of those used for the dissection gesture classification task (Table 2, right).

We hypothesized that this task of phase recognition (distinguishing between nerve-sparing and suturing) is quite trivial to achieve. This is because the visual cues of each activity are markedly distinct from one another. Indeed, we found that SAIS reliably distinguished between these two activities as evident by its AUC = 1 (Fig. 2).

Fold	Training		Validation		Testing	
	n	v	n	v	n	v
0	2472	148	240	23	372	28
1	2616	144	180	23	228	28
2	2544	147	192	24	300	28
3	2448	147	156	23	396	28
4	2604	143	240	24	240	28
5	2352	147	264	25	444	27
6	2604	147	468	24	240	28
7	2652	148	252	26	192	28
8	2604	145	396	25	240	27
9	2352	147	204	25	432	29

Supplementary Table 5. Total number of videos samples (n), videos (v), and surgeons (s) in the training, validation, and test sets of each fold for phase recognition. These data splits are used for the 10-fold Monte Carlo cross-validation.

.

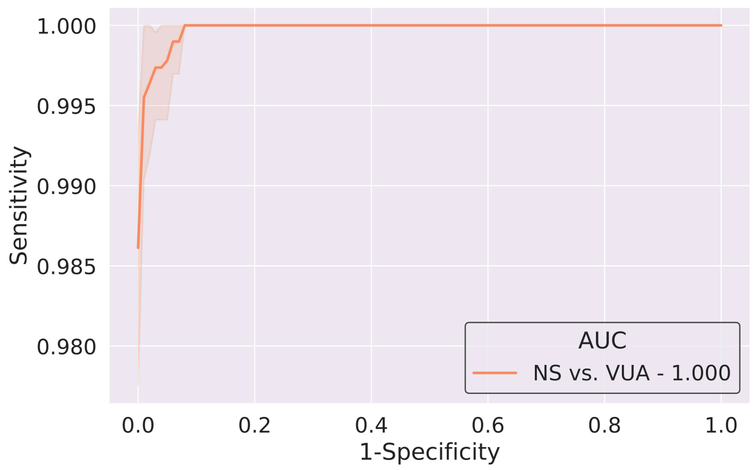

Supplementary Figure 2. **SAIS reliably decodes surgical phases across videos.** SAIS is trained on video samples exclusively from USC and also evaluated on video samples from USC. Results are shown as an average (±1 standard deviation) of 10 Monte-Carlo cross-validation steps.

Supplementary Note 4 - Validating SAIS on external datasets

We validated SAIS on two external datasets: JIGSAWS suturing and DVC UCL, and compared its performance to that of state-of-the-art methods for these respective datasets (see Supplementary Table 6 and 7). We report these results in the main manuscript (see Results). In short, we find that SAIS, despite not being purposefully designed for these datasets, performs competitively with the baseline methods.

Method	Accuracy	Modalities
Fusion-KV[1]	86.3	Video + Kinematics
MS-RNN[2]	90.2	Kinematics
Sym. Dilation[3]	90.1	Video
SAIS (ours)	87.5 (13.0)	Video

Supplementary Table 6. **Accuracy of gesture classification on the JIGSAWS suturing dataset.** We report the accuracy of the best-performing methods[4] evaluated using leave-one-user-out (LOUO) cross-validation and in each modality category.

Method	Accuracy (%) Random	Reported	Improved
MA-TCN[5]	25.9	80.9	3.1×
SAIS (ours)	14.3	59.8 (1.0)	**4.2**×

Supplementary Table 7. **Accuracy of gesture classification on the DVC UCL dataset.** MA-TCN reports accuracy on a private test-set with gesture imbalance. We report the average cross-validation accuracy on the publicly-available training set with balanced categories. Bold indicates the better-performing method.

References

1. Qin, Y. *et al.* Temporal segmentation of surgical sub-tasks through deep learning with multiple data sources. In *2020 IEEE International Conference on Robotics and Automation (ICRA)*, 371–377 (IEEE, 2020).

2. Gurcan, I. & Van Nguyen, H. Surgical activities recognition using multi-scale recurrent networks. In *ICASSP 2019-2019 IEEE International Conference on Acoustics, Speech and Signal Processing (ICASSP)*, 2887–2891 (IEEE, 2019).

3. Zhang, J. *et al.* Symmetric dilated convolution for surgical gesture recognition. In *International Conference on Medical Image Computing and Computer-Assisted Intervention*, 409–418 (Springer, 2020).

4. van Amsterdam, B., Clarkson, M. & Stoyanov, D. Gesture recognition in robotic surgery: a review. *IEEE Transactions on Biomed. Eng.* (2021).

5. Van Amsterdam, B. *et al.* Gesture recognition in robotic surgery with multimodal attention. *IEEE Transactions on Med. Imaging* (2022).
