# Supplementary material for: A vision transformer for decoding surgeon activity from surgical videos
Source: Nat Biomed Eng. 2023 Mar 30;7(6):780–96. doi: 10.1038/s41551-023-01010-8 (PMC10307635; doi:10.1038/s41551-023-01010-8)
Supplement: Supplementary file 1 [file 41551_2023_1010_MOESM2_ESM.pdf]

nature research

Corresponding author(s): Dani Kiyasseh

Last updated by author(s): 2023/01/13

Reporting Summary

Nature Research wishes to improve the reproducibility of the work that we publish. This form provides structure for consistency and transparency in reporting. For further information on Nature Research policies, see our Editorial Policies and the Editorial Policy Checklist.

Statistics

For all statistical analyses, confirm that the following items are present in the figure legend, table legend, main text, or Methods section.

n/a	Confirmed	
☐	☒	The exact sample size (n) for each experimental group/condition, given as a discrete number and unit of measurement
☐	☒	A statement on whether measurements were taken from distinct samples or whether the same sample was measured repeatedly
☒	☐	The statistical test(s) used AND whether they are one- or two-sided<br>*Only common tests should be described solely by name; describe more complex techniques in the Methods section.*
☐	☒	A description of all covariates tested
☒	☐	A description of any assumptions or corrections, such as tests of normality and adjustment for multiple comparisons
☐	☒	A full description of the statistical parameters including central tendency (e.g. means) or other basic estimates (e.g. regression coefficient) AND variation (e.g. standard deviation) or associated estimates of uncertainty (e.g. confidence intervals)
☒	☐	For null hypothesis testing, the test statistic (e.g. F, t, r) with confidence intervals, effect sizes, degrees of freedom and P value noted<br>*Give P values as exact values whenever suitable.*
☒	☐	For Bayesian analysis, information on the choice of priors and Markov chain Monte Carlo settings
☒	☐	For hierarchical and complex designs, identification of the appropriate level for tests and full reporting of outcomes
☒	☐	Estimates of effect sizes (e.g. Cohen's d, Pearson's r), indicating how they were calculated

Our web collection on statistics for biologists contains articles on many of the points above.

Software and code

Policy information about availability of computer code

Data collection	No software was used to collect data.
Data analysis	Custom code, Python 3, PyTorch 1.8.0. Code is available at https://github.com/danikiyasseh/SAIS.

For manuscripts utilizing custom algorithms or software that are central to the research but not yet described in published literature, software must be made available to editors and reviewers. We strongly encourage code deposition in a community repository (e.g. GitHub). See the Nature Research guidelines for submitting code & software for further information.

Data

Policy information about availability of data

All manuscripts must include a data availability statement. This statement should provide the following information, where applicable:

- Accession codes, unique identifiers, or web links for publicly available datasets
- A list of figures that have associated raw data
- A description of any restrictions on data availability

The data supporting the results in this study involve surgeon and patient data. The data from St. Antonius Hospital and Houston Methodist Hospital are not publicly available, yet de-identified data from the University of Southern California can be made available by the corresponding authors on reasonable request.

Field-specific reporting

Please select the one below that is the best fit for your research. If you are not sure, read the appropriate sections before making your selection.

☒ Life sciences ☐ Behavioural & social sciences ☐ Ecological, evolutionary & environmental sciences

For a reference copy of the document with all sections, see nature.com/documents/nr-reporting-summary-flat.pdf

Life sciences study design

All studies must disclose on these points even when the disclosure is negative.

Sample size	All experiments were conducted using 10-fold Monte Carlo cross-validation. Details of the number of samples and surgical videos used during training, validation and testing are provided in the Supplementary Information.
Data exclusions	We did not exclude any patients for training, validation or testing.
Replication	The protocol is described in the paper, and in-depth implementation details are provided in the Supplementary Information.
Randomization	The experiments were conducted across 10 folds. Each fold consisted of a different subset of data on which to train and evaluate the model. The initialization of the parameters of the network was also different across folds. Each fold was set based on a seed (from 0 to 10).
Blinding	Blinding was not applicable. When comparing different methods, we maintained the same experimental settings to allow for a fair comparison.

Reporting for specific materials, systems and methods

We require information from authors about some types of materials, experimental systems and methods used in many studies. Here, indicate whether each material, system or method listed is relevant to your study. If you are not sure if a list item applies to your research, read the appropriate section before selecting a response.

Materials & experimental systems

n/a	Involved in the study	
☒	☐	Antibodies
☒	☐	Eukaryotic cell lines
☒	☐	Palaeontology and archaeology
☒	☐	Animals and other organisms
☐	☒	Human research participants
☒	☐	Clinical data
☒	☐	Dual use research of concern

Methods

n/a	Involved in the study	
☒	☐	ChIP-seq
☒	☐	Flow cytometry
☒	☐	MRI-based neuroimaging

Human research participants

Policy information about studies involving human research participants

Population characteristics	Table 1 provides a summary of the number of video samples used for the different experimental tasks (sub-phase recognition, gesture classification, and skill assessment). We also include the total number of surgeons from each hospital who were associated with these samples.
Recruitment	Surgeons from different hospitals were recruited as part of Award No. R01CA251579-01A1 by the National Cancer Institute. Videos from hospitals exclusively reflected two different types of surgical procedures: robot-assisted radical prostatectomy and robot-assisted partial nephrectomy. Details of these procedures can be found in Methods.
Ethics oversight	All datasets (data from the University of Southern California, St. Antonius Hospital, and Houston Methodist Hospital) were collected under Institutional Review Board (IRB) approval, in which informed consent was obtained (HS-17-00113). These datasets were de-identifed prior to model development.

Note that full information on the approval of the study protocol must also be provided in the manuscript.
